# Parathyroid adenoma presenting as genu valgum in a child: A rare case report

**DOI:** 10.1016/j.ijscr.2019.03.063

**Published:** 2019-04-09

**Authors:** K. Sreedhar Rao, Praveen Agarwal, Jayachandra Reddy

**Affiliations:** Department of General Surgery, Kamineni Hospitals, Hyderabad, India

**Keywords:** Parathyroid adenoma, Genu valgum, Hyperparathyroidism, Technetium-99m sestamibi, Case report

## Abstract

**Introduction:**

Parathyroid adenoma is the most common cause of primary hyperparathyroidism (PHPT). Patients typically present with elevated serum calcium levels and elevated serum parathyroid hormone levels. The incidence and prevalence is higher in adults but is rare in pediatric age group.

**Case presentation:**

A 12-year-old, pre-pubertal female presented with pain in bilateral knee joints and gait abnormality since one year. Her past medical history and family history were insignificant. On general examination, no other congenital bony deformities were observed besides genu valgum. Laboratory tests showed hypocalcemia and elevated serum levels of intact parathyroid hormone. Radiograph of bilateral both knee joints was suggestive of genu valgum. Ultra sonogram of neck revealed enlarged parathyroid gland and a Technetium (99mTc) sestamibi scan was suggestive of right inferior parathyroid adenoma. Surgical resection was performed and post-operative hypocalcemia was seen. After necessary treatment was given and the patient was discharged when asymptomatic.

**Discussion and conclusion:**

Parathyroid adenoma causing primary hyperparathyroidism is a rare disease in children. They typically present with nonspecific symptoms involving gastrointestinal, musculoskeletal, renal and neurological symptoms due to hypercalcemia. Moreover, Genu valgum is a rare presentation in children with parathyroid adenoma.

## Highlights

• Primary hyperparathyroidism is a rare disease in children and adolescents.
• Genu valgum is a rare presentation in children with PHPT.
• Primary hyperparathyroidism may present as normocalcemic primary hyperparathyroidism if associated with vit. D deficiency.
• Technetium99 M-sestamibi scan is most sensitive and specific for localization of parathyroid adenoma.
• Surgery is the only curative treatment for PHPT and post-operative management for features of hypocalcemia is crucial.

## 1 Introduction

Parathyroid adenoma is the most common cause of primary hyperparathyroidism (PHPT). Parathyroid adenoma is part of a spectrum of parathyroid proliferative disorder that includes parathyroid hyperplasia, parathyroid adenoma, and parathyroid carcinoma. Patients typically present with elevated serum calcium levels and elevated serum parathyroid hormone levels. PHPT is caused by parathyroid adenoma followed by primary parathyroid hyperplasia and parathyroid carcinoma in 80–85 percent of cases [1]. The estimated incidence of PHPT in pediatric patients is 1 per 200–300,000 and its prevalence is 2–5 in 100,000 [2] with no difference in distribution by sex [3] with a higher predominance in adolescents [4].

We present a rare case of parathyroid adenoma with PHPT presenting as Genu valgum, which was diagnosed and treated, at our hospital and this work has been reported in line with the SCARE criteria [5].

## 2 Presentation of case

A 12-year-old, pre-pubertal female presented with pain in bilateral knee joints and gait abnormality since one year was referred to the department of general surgery for evaluation of parathyroid abnormality. Her past medical history did not reveal any history of trauma, fractures, abdomen pain, anorexia, vomiting, constipation, headache, blurred vision, polyuria, fatigue, depression, irritability and renal stones. Her family history did not of reveal any endocrine or skeletal disorders. On general examination besides genu valgum, she had no other congenital bony deformities. There was no palpable mass in the neck on physical examination.

**Fig. 1:**
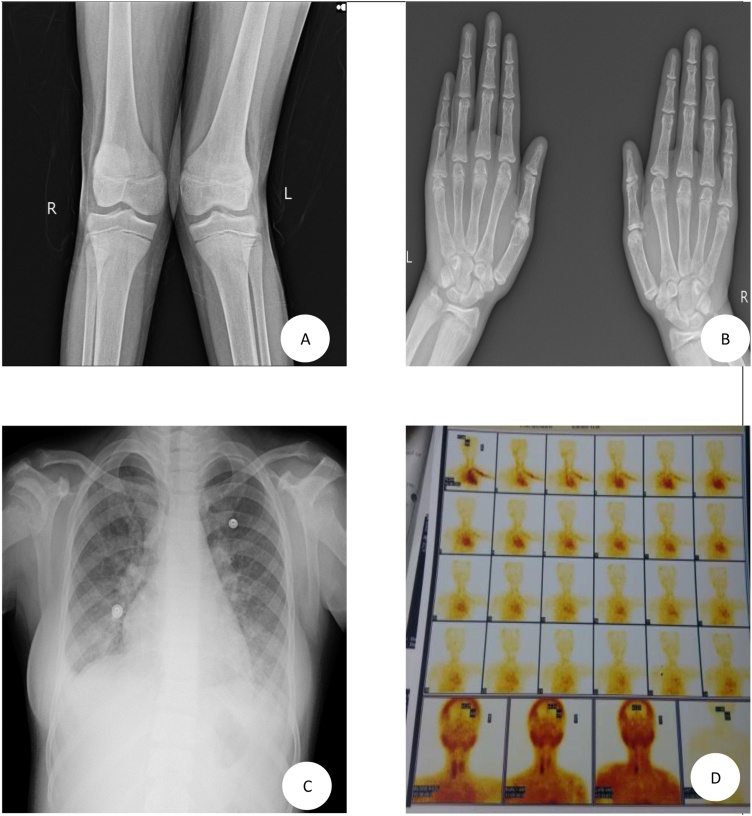
A: Radiograph bilateral both knee suggestive of genu valgum. B: radiograph bilateral both hand suggestive of acro-osteolysis. C: radiograph chest PA view suggestive of subchondral resorption on the distal ends of the clavicle. D: Tc 99 m Sestamibi scan suggestive of right inferior parathyroid adenoma.

Laboratory tests showed mild anemia (10.2 g/dL; normal range: 11.–14.0 g/dl); Serum calcium (8.1 mg/dl; normal range: 8.4–10.4 mg/dl), serum phosphorous (2.8 mg/dL; normal range, 2.6–4.4 mg/dL); decreased 1, 25-dihydroxy vitamin D (17.79 ng/ml; normal range: 30–100 ng/mL), elevated alkaline phosphatase (6690 IU/L; normal range: 42–98 IU/L), elevated serum levels of intact parathyroid hormone (iPTH) (2616 pg/mL; normal range, 10–65 pg/mL). Radiograph of bilateral both knee joints was suggestive of genu valgum (Fig. 1A). Ultra sonogram of neck revealed cystic changes in the right inferior parathyroid gland with lobulations, the size of gland being 32-x40 mm. Ultra sonogram of Kidney, ureter and bladder suggested of normal study. A skeletal survey (Fig. 1B & C) revealed generalized osteopenia, subchondral resorption on the distal ends of the clavicle and acro-osteolysis of distal phalanges. A Technetium (99mTc) sestamibi scan was suggestive of right inferior parathyroid adenoma (Fig. 1D). Patient was also screened for MEN-I and MEN-II syndrome. A diagnosis of Parathyroid adenoma was made and was scheduled for resection.

**Fig. 2:**
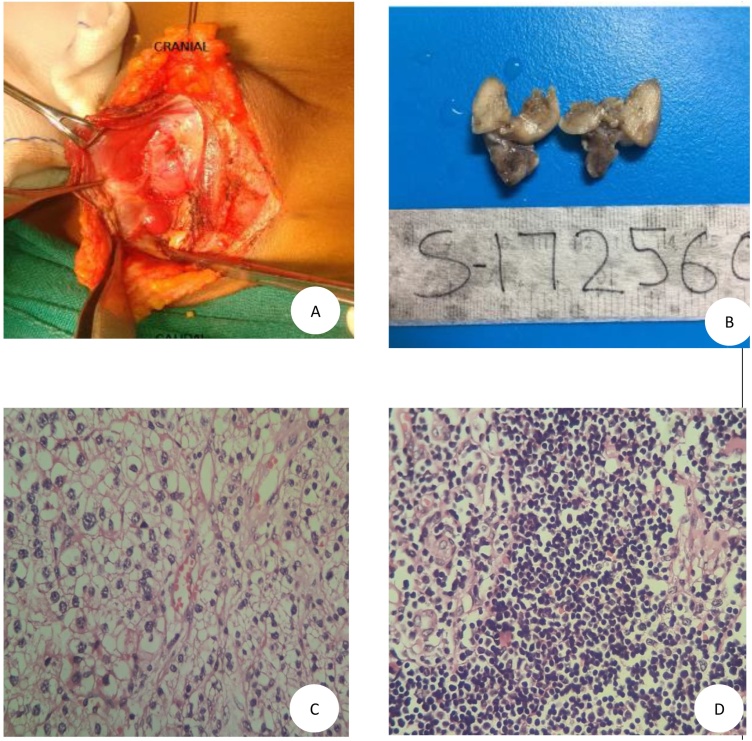
A: Bean shaped nodule partly embedded in lower pole of right lobe of thyroid. B: Gross specimen of parathyroid. C: Histopathology of parathyroid gland showing encapsulated, cellular, homogenous lesions composed of chief cells in delicate capillary network. D: Histopathology of thymus.

After preoperative evaluation, she underwent parathyroidectomy under general anesthesia, which was identified as a bean shaped nodule partly embedded in lower pole of right lobe of thyroid peri-operatively (Fig. 2A). The tumor was resected from lower pole of right lobe of thyroid along with thymus. Macroscopically, the tumor was a solid, yellowish-brown mass measuring 30 × 20 mm, with partial foci of hemorrhage. On gross examination, the resected specimen was well circumscribed, enclosed by a thin, fibrous, capsule-like structure. The tumor was with few small nodules with surrounding adipose tissue (Fig. 2B). The surgical procedure was uneventful. Histopathology of the specimen revealed encapsulated, cellular, homogenous lesions composed of chief cells in delicate capillary suggestive of parathyroid adenoma (Fig. 2C). However, the histopathology of thymus was normal (Fig. 2D).

Post-operatively, the patient experienced perioral numbness and carpo-pedal spasm on the night of surgery. Initial clinical examination revealed a positive Chvostek sign. Laboratory investigations revealed total calcium (9.2 mg/dl; normal range: 8.4–10.4 mg/dl), ionized calcium (3.8 mg/dl) and serum phosphate (1.9; normal range, 2.6–4.4 mg/dL). The electrocardiogram was within normal limits. She was treated for hypocalcemic tetany with Injection 10% Calcium Gluconate, 10 ml over 10 min as a bolus and then with calcium gluconate infusion at 1 mg/kg/hr for which total recovery was observed. The same treatment continued for two more days. The infusion was weaned on the third post-operative day and treatment was continued with oral elemental calcium 500 mg TID. Laboratory parameters were within normal limits. The patient recovered well symptomatically and was discharged on fifth post-operative day.

## 3 Discussion

In this case, we treated a 12-year-old female patient with PHPT caused by parathyroid adenoma. Parathyroid adenoma is a benign encapsulated neoplasm of parathyroid gland usually involving single gland mostly composed of chief cells, but mixture of oncocytic cells, transitional oncocytic cells can also be seen [4]. PHPT occurs in adults and is rare in children [6,7]. It can occur as a component of MEN type I and II in pediatric patients with PHPT presenting with nonspecific symptoms like anorexia, nausea, vomiting, constipation, irritability, polyuria and polydipsia. Patients may also present with nephrolithiasis, bone pain, rickets or acute pancreatitis [8]. Osseous complications may rarely be the first symptom of hyperparathyroidism. Hence, there could be delay in diagnosis of PHPT in children and is sometimes missed. Osseous changes owing to hypercalcemia are more common in children than in adults and may lead to pain in the back or extremities, gait disturbances, pathological fractures or genu valgum as observed in this case. Genu valgum is a rare presentation in children with PHPT with only few cases reported in literature [9]. Hypercalcemia as a result of excessive secretion of PTH is considered as the hallmark of PHPT [10].

In our case, we found the patient’s PTH levels to be extremely high with mild hypocalcemia, normal phosphorous levels and low vit. D level (17.79 ng/ml). If patients have levels of 25-hydroxyvitamin D below 30 ng/mL, a normal serum calcium can be associated with elevated PTH [11]. If low serum vitamin D causes elevation in PTH, repletion with vitamin D would normalize PTH level [11]. It should be noted, however, that occasionally when vitamin D deficiency is corrected, a normocalcemic patient becomes hypercalcemic and thus their presentation becomes that of traditional hypercalcemic primary hyperparathyroidism. In that instance, the normocalcemia was due to the Vitamin D deficiency [11].

The patient did not have any symptoms at presentation other than bilateral genu valgum neither did she have any family history of parathyroid disorders. The Technetium (99mTc) sestamibi uptake scan demonstrated focal increase in uptake of tracer in the right inferior lobe of the thyroid suggesting parathyroid adenoma for which she underwent surgical removal of the tumor. Post-operatively patient developed symptomatic hypocalcemia. Preoperative normocalcemia was significantly associated with increased risk of late biochemical hypocalcemia. preoperative normocalcemia of <11 was associated with an increased risk of early postoperative hypocalcemia but not with late hypo-calcemia. Treatment of severe HBS after parathyroidectomy requires intravenous calcium administration under electrocardiographic monitoring, high dose activated vitamin D, intravenous magnesium sulphate or oral magnesium oxide in the case of deficiency. The patient recovered well before discharge.

## 4 Conclusion

Parathyroid adenoma causing primary hyperparathyroidism is a rare disease in children. They typically present with nonspecific symptoms involving gastrointestinal, musculoskeletal, renal and neurological symptoms due to hypercalcemia. Preoperatively primary parathyroidism may be associated with normocalcemia if there is vit. D deficiency. Moreover, Genu valgum is a rare presentation in children with parathyroid adenoma. Ultra sonogram and Technetium (99mTc) sestamibi scan are useful in localizing the parathyroid adenoma. Though the exact mechanism of genu valgum in these children with primary hyperparathyroidism needs to be elucidated, it is proposed that elevated parathyroid hormone levels may have a direct effect on the growth plates during pubertal growth spurt resulting in genu valgum [12]. Surgery is the only curative treatment for parathyroid adenoma and post-operative management for features of hypocalcemia is crucial.

## Conflicts of interest

No conflicts of interest declared by the authors.

## Sources of funding

No funding has been declared by the authors.

## Ethical approval

“Institutional Ethics Committee of Kamineni Hospitals” approved this publication of this case report (Registration #: ECR/58/Inst/AP/2013/RR-16).

## Consent

Written informed consent was obtained from the patient’s parents as patient is minor, for publication of this case report and accompanying images. A copy of the written consent is available for review by the Editor-in-Chief of this journal on request.

## Author contribution

Dr. Praveen Agarwal: Evaluation and post-operative management of the case along with surgical assistance.

Dr. K. Sreedhar Rao: Performed the surgical technique.

Dr. Jayachandra Reddy: Assisted the surgical procedure.

## Registration of research studies

This is a case report. Hence, it is not registered in the clinical trial registry.

## Guarantor

Dr. K. Sreedhar Rao.

## Provenance and peer review

Not commissioned, externally peer-reviewed.
